# Puricelli biconvex arthroplasty: an experimental study in sheep

**DOI:** 10.1186/s13005-023-00379-w

**Published:** 2023-08-02

**Authors:** Renan Langie, Viviane Neves Pacheco, Vinicius Matheus Szydloski, Adriana Corsetti, Alexandre Silva de Quevedo, Fábio Pinto da Silva, Fabrício Mezzomo Collares, Fernanda Visioli, Deise Ponzoni, Edela Puricelli

**Affiliations:** 1 Department of Surgery and Orthopedics, School of Dentistry / Unit of Oral and Maxillofacial Surgery, Clinics Hospital of Porto Alegre – University Hospital, Federal University of Rio Grande Do Sul (UFRGS), Porto Alegre, RS Brazil; 2 Department of Oral and Maxillofacial Pathology, School of Dentistry, Federal University of Rio Grande Do Sul (UFRGS), Porto Alegre, RS Brazil; 3 Department of Oral and Maxillofacial Surgery, Santa Casa de Misericórdia de Porto Alegre (SCMPA), Porto Alegre, RS Brazil; 4 Department of Oral and Maxillofacial Surgery, Düsseldorf University Hospital (UKD), Henrich-Heine University (HHU), Düsseldorf, NRW Germany; 5 Laboratory of Design and Material Selection (LDSM), School of Engineering, Federal University of Rio Grande Do Sul (UFRGS), Porto Alegre, RS Brazil; 6 Laboratory of Dental Materials (LAMAD), School of Dentistry, Federal University of Rio Grande Do Sul (UFRGS), Porto Alegre, RS Brazil

**Keywords:** Temporomandibular joint, Temporomandibular joint disorders, Arthroplasty, Maxillofacial reconstruction, PMMA, Sheep

## Abstract

**Background:**

The aim of this study was to establish a sheep model of the Puricelli biconvex arthroplasty (ABiP) technique in sheep for evaluating its functional, biological and histological parameters.

**Methods:**

Ten Corriedale black sheep were submitted to TMJ total reconstruction with poly(methyl methacrylate) (PMMA) using ABiP and euthanized after 45 (*n* = 5) or 90 (*n* = 5) days. Control animals (*n* = 2) underwent sham operations and were euthanized after 45 days. Variables were assessed before the surgery (T0), immediately after (T1) and at 45 or 90 postoperative days (T2).

**Results:**

Histological analyses showed regression of inflammatory cells over the follow-up period. PMMA showed reduced porosity and roughness in the articular contact area. PMMA temporal components showed linear and volumetric wear in comparison to control, but no foreign body reaction was observed. The reconstructions were stable in all animals. The amplitude of mouth opening and left lateral movements were maintained, except for a reduction in the range of right lateral movements at day 90 in the experimental group. Clinical, macroscopic and radiographic observations showed that the reconstructions were stable.

**Conclusions:**

The analysis of functional, biological and histological parameters in sheep submitted to ABiP showed stable results of the procedure, with maintenance of body weight and all mandibular movements, save contralateral mandibular movement, suggesting that joint function was completely maintained following the procedure. This experimental study provides support for clinical results previously reported of the ABiP technique in TMJ reconstruction procedures.

## Introduction

The pathologies that affect the temporomandibular joint (TMJ) compose a heterogeneous group of conditions with multifactorial aetiologies and difficult treatment courses. Conservative and clinical therapies are usually the first treatment option for such conditions. However, in unresponsive cases, and in the presence of proliferative or degenerative joint disease, surgical interventions may be recommended aimed at structural and functional recovery of the joint [1]. The prevalence of bone disorders is rising due to a global aging population, consequently the demand for TMJ total reconstruction is increasing [2].

The continuing search for improvement in surgical techniques, materials and protocols reflects the difficulty in obtaining a satisfactory method for TMJ reconstruction [3]. Instability of the reconstruction and inability to bear articular loads have been reported as the main causes of failure following total joint reconstruction [4].

Acrylic bone cements, polymer-ceramic composites based on poly(methyl methacrylate) (PMMA), have been widely used in joint replacements for decades. As reviewed by Bota et al. [5], the history of the use of PMMA as an articulating surface in orthopaedics dates from 1941, when Judet described an acrylic hemiarthroplasty in hip surgery. Scales and Zarek [6] studied the Judet hip prosthesis both biomechanically and clinically and concluded that the technique, and acrylic in particular, were inappropriate for use in hip arthroplasty. Other limitations were described for PMMA, related to the possibility of bone cement implantation syndrome (BCIS) due to the release of methyl methacrylate monomer (MMA) into the bloodstream [7–9], or damage to surrounding tissues due to exothermic reaction during the PMMA polymerization process [10]. These limitations can be overcome, however, by appropriate manipulation of the material [11, 12], so the use of PMMA in joint reconstruction is still current, not only in hip metallic prosthesis but also in vertebroplasty and kyphoplasty [13], and in craniofacial surgery such as orbital and cranial reconstruction [14, 15].

PMMA is also used in the Puricelli biconvex arthroplasty (ABiP) technique, employed in temporomandibular joint reconstruction in adult and paediatric patients [16, 17] and recently described in greater detail [18]. In ABiP, two PMMA components with minimal, stable contact between the convex surfaces are used for TMJ replacement. This low-cost, more conservative surgery, with single access and limited ostectomy, results in adequate joint stability and dental occlusion, and long-term follow-up (up to 43 years) [18] has shown no joint noises, pain or movement restrictions in patients. Despite excellent clinical results, there is a lack of experimental studies for the investigation of biological and histological parameters of ABiP.

Large animal models are critical for the establishment and adequate understanding of therapeutic approaches. The sheep is considered a valid model for TMJ studies, due to TMJ characteristics such as size, shape of the condylar process and mandibular fossa and disc size [19, 20]. As ruminants, their masticatory function is also an important characteristic for research of TMJ techniques. The present study aims to establish a model of ABiP in sheep, to evaluate the reconstruction capsule and pseudo-disc regarding inflammatory response, the degree of polymer conversion, roughness, wear and hardness of the acrylic prosthetic components, as well as to further characterize its surface through scanning electron microscopy (SEM). Functional parameters, including joint mobility and change in body weight, have also been investigated.

## Material and methods

### Pilot study

A surgeon was trained using two sheep skulls: one refrigerated (with soft tissues still attached) and one dried (only hard tissues present). The surgeon was required to access the TMJ, perform a condylectomy, wax the two articular surfaces in situ, take an impression of the structures, obtain plaster models, and create 1 mm-thick silicone trays of the temporal and mandibular segments. These trays were used later in the study.

Aiming to draw comparisons with the acrylic components (mandibular and temporal) removed from the animals in the experimental period of 45 days (test samples), acrylic control components (C0) with the same preparation and dimension were created, but not exposed to the biological environment. Specific control samples were prepared for assessing the PMMA degree of conversion.

### Sample allocation and study design

Sample size was calculated in accordance with the literature [21, 22]. Fourteen young adult (ten to fourteen months old) female Corriedale black sheep, weighing approximately 35 kg, were used in the present study. The sheep were divided into an experimental (E) and a control group (C) through unequal randomization (ratio 5:2). The animals in the experimental group (*n* = 10) underwent biconvex arthroplasty, and were further divided into two subgroups, E45 (*n* = 5) and E90 (*n* = 5), according to the interval between the surgical procedure and euthanasia (45 and 90 days, respectively). Control animals (C45, *n* = 2) underwent pre-, intra- and postoperative procedures, but surgery was limited to skin incision and subcutaneous and muscular dissection without approaching the joint capsule or bone. Variables were assessed before the surgery (T0), immediately after (T1) and at 45 or 90 postoperative days (T2). All surgical procedures were conducted by the same surgeon.

### Pre-operative care and anaesthetic procedures

Pre-operative care involved transporting, weighing and housing the animals for 24 h at the Animal Experimental Unit, with a view to reducing stress. The animals were submitted to a 24-h fast from solid foods and a 12-h fast from liquids. Housing temperature was kept at 22 °C ± 2 °C, humidity was maintained at 60% ± 5 and the light/dark cycle was controlled by a timer (12:12, lights on at 7 h and off at 19 h).

The pre-anaesthetic medications meperidine (3 mg/kg) and midazolam (0.25 mg/kg) were administered intramuscularly (IM). After 15 min, the animals were contained and transported to the operating room. Cefazolin (22 mg/kg) was administered intravenously, as was the anaesthetic propofol (3–5 mg/kg). The animals were submitted to orotracheal intubation followed by mechanical ventilation and isoflurane anaesthesia.

### Surgical procedures

**Fig. 1:**
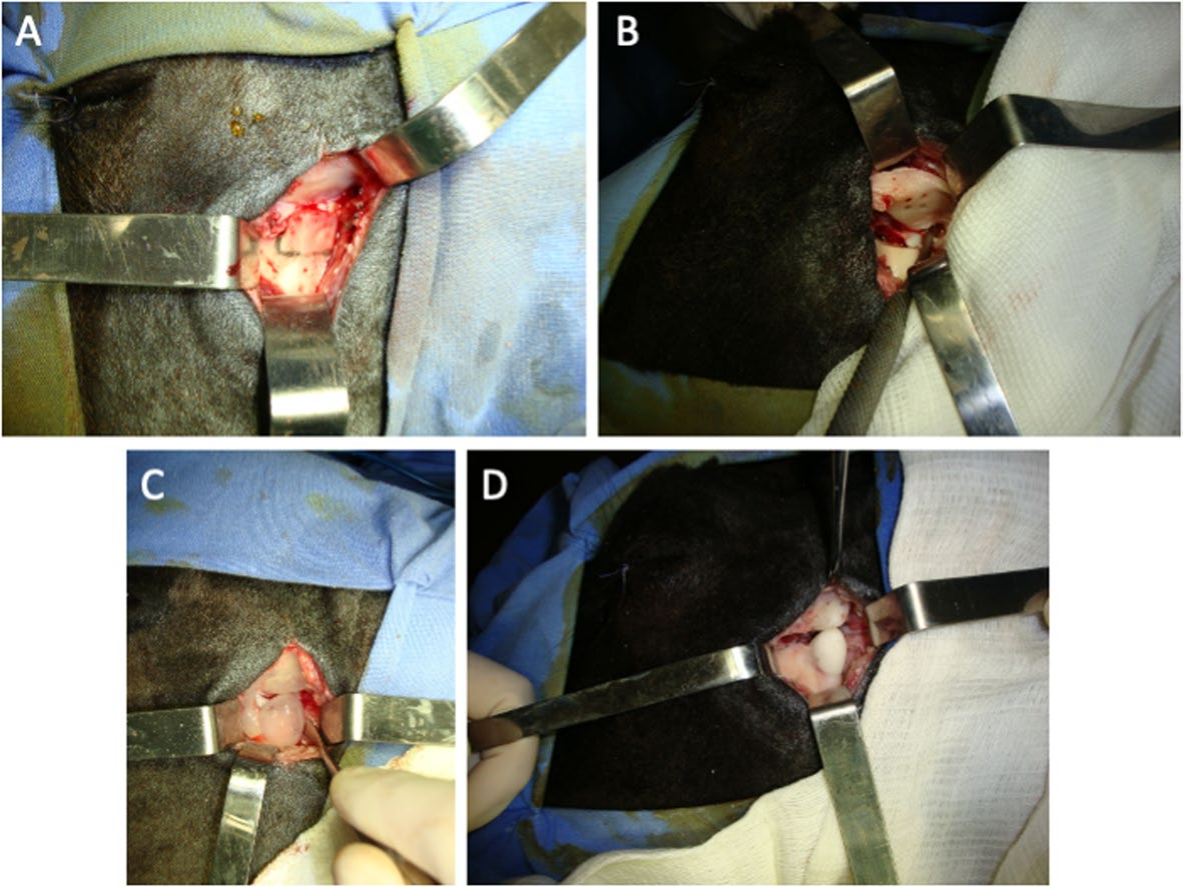
TMJ reconstruction using the Puricelli biconvex arthroplasty technique. **A** Condylectomy; **B** Temporal bicortical perforations; **C** Silicone trays of the temporal and mandibular segments before the reconstruction; **D** Final reconstruction of the left TMJ

A trichotomy and antisepsis were performed and surgical drapes were positioned. A preauricular incision (7 cm) and tissue dissection/detachment were performed to access the TMJ. A line was drawn from the mandibular condyle to the temporal bone to define the orientation of the jaw force axis (posterior-superior vector, 45^o^ from the occlusal plane). This line was used to identify the future location of the contact point between the reconstructed segments, which allows for the jaw force vector to be directed downwards. The new mandibular condyle, which is articulated with the posterior surface of the glenoid cavity, guides the vector on a sagittal plane, directing it in an inferior to superior and anterior to posterior direction. An anterior–posterior osteotomy was performed in the condylar neck, 10 mm from the highest point of the condyle (Fig. 1A). Four and three bicortical perforations (1 mm) were made on the temporal surface (Fig. 1B) and condylar neck, respectively, for mechanical retention of the PMMA cement, since the material has no adhesive property to the bone. At this point, the animal was submitted to maxillomandibular immobilization. Auto-polymerized poly(methyl methacrylate) (Surgical Simplex P Bone Cement, Howmedica International Inc, Limerick, Ireland) was mixed according to the manufacturing company’s instructions and injected into the bone perforations. The temporal silicone tray (Fig. 1C) was also completely filled. As the material hardened, the tray was placed in position. During the heating phase, the area was constantly irrigated with distilled water. After the polymerization, the silicone tray was opened with a scalpel and removed. The same sequence was performed in the mandibular segment. When the reconstruction was completed (Fig. 1D), intermaxillary immobilization was removed. Retention of the alloplastic implants and maintenance of mandibular mobility were confirmed through manual examination of the mouth-opening amplitude and of lateral mandibular movements. The incision was closed using a simple interrupted suture with polyglactin 910 thread.

In the animals of the control group, a vertical preauricular 7-cm incision was made, followed by incision and divulsion of the masseteric musculature. In this group, the joint capsule was not ruptured, so the joint surfaces were not exposed and the procedures for joint reconstruction were not performed. The tissue planes were sutured with 4 × 0 polyglactin 910.

### Postoperative management

Animals began to be fed immediately after recovering from the anaesthesia. After 48 h, the sheep were transferred to external housing, where they were identified by ear tags on both ears. A broad-spectrum antimicrobial agent (benzylpenicillin/streptomycin, 6 ml/100 kg) was administered IM following surgery, and once again after 72 h. Anti-inflammatory medication (ketoprofen, 2 mg/kg) was also administered daily for 48 h, while an analgesic (tramadol hydrochloride, 2 mg/kg) was given to the animals every 12 h for 48 h.

Experimental and control animals were returned to the Animal Experimental Unit after 45 or 90 days. After their body weight had been measured, mouth-opening and mandibular laterality amplitude were assessed under general anaesthesia. Following the evaluation of these parameters, the anaesthetized animals were euthanized using intravenous potassium chloride (1 ml/kg).

The reconstructed TMJ was surgically exposed post-mortem and the left temporomandibular joint was removed with a 6.0 cm (diameter) × 4.5 cm (depth) trephine drill. The samples were identified and stored in closed containers with 10% buffered formalin.

### Histological analyses

After fixation, samples from E45 and E90 animals were decalcified in 5% nitric acid solution for approximately two weeks. Decalcification allowed the removal of prosthetic components from bone tissue without damage. Samples from the reconstruction capsule and pseudo-disc were collected from the main block, identified and submitted to routine histological processing before being embedded in paraffin. Slides were stained using hematoxylin–eosin (HE). Morphological descriptive analysis of the capsule and pseudo-disc samples (when present) in HE staining at 100 × magnification was performed using a CX41RF binocular microscope (Olympus Latin America Inc., Miami, FL, USA). Images of five selected fields from the most cellular areas of the samples under investigation were captured at 400 × magnification using a Qcolor 5, Coolet, RTV camera (Olympus Latin America Inc.) and Qcapture software version 2.81 (Quantitative Imaging Corporation Inc., Canada). All inflammatory cells were quantified using ImageJ for Windows version 3.0 software (NIH, USA) in the five fields and classified into neutrophils, eosinophils, lymphocytes, plasmocytes and macrophages. Data are presented as mean and standard deviation considering each animal as sample. A blinded, trained and calibrated examiner performed quantifications. For calibration purposes, 30 fields from six different samples were evaluated, and re-evaluated after five days, and quantifications were compared using the Intraclass Correlation Test (ICC) (α > 0.98).

### PMMA degree of conversion

Vibrational Raman spectroscopy (Senterra, BrukerOptics, Germany) was performed at three random points in the areas corresponding to the contact point established between the temporal and condylar PMMA surfaces, the non-contact area between the components and the contact surface of the acrylic with the bone tissue, in order to evaluate the degree of conversion of the acrylic resin. The 100 mW and 785 nm diode laser was used for two seconds with 20 co-additions, totalling 40 s with 100% laser power, and a spectral resolution of 3–5 cm^−1^. Spectra were obtained between 400 and 1800 cm^−1^.

The percentage of unreacted carbon–carbon double bonds (%C = C) was determined by the ratio of the absorbances between the aliphatic carbon double bonds (peak at 1640 cm^−1^) and the internal standard in the monomer and the polymer. The absorbance of the carbonyl group (peak at 1720 cm^−1^) was used as an internal standard. The degree of conversion (DC) was determined by subtracting the %C = C of 100% (13). For this analysis, control specimens (*n* = 3) were made using a silicone matrix 2 mm in height and 3 mm in diameter. A spectrum was obtained soon after the manipulation of the material and insertion into the matrix to obtain the values referring to the peaks in the monomer. The same specimens were analysed immediately after the cure time (ImmC) and after seven days (7dC). The relative values obtained for the monomer were also used to calculate the degree of conversion of the components.

### PMMA roughness assessment

An SJ-201 rugosimeter (Mitutoyo, Japan) was used to assess the components’ surface roughness. It contains a sensor which, when traversing the surface of the material, assigns values that define peaks and valleys present on this surface. The value assigned to the area of peaks and valleys was divided by the distance travelled by the straight-line sensor providing the roughness parameter in R_a_ in μm. The machine provides the average of three polls of 0.25 μm. Four measurements were performed randomly, by a blinded examiner, on each surface (with without wear and control C0) on the condylar and temporal samples.

### PMMA scanning electron microscopy

The samples were metallized by a gold film using an ion deposition technique (sputter coater) due to the absence of electrical conduction of the acrylic material. Afterwards, they were stabilized in stubs and scanned with an electron microscope (SEM-FEG) (Inspect F50, FEI, Czech Republic). Images of the surfaces with or without wear, the acrylic contact area with the bone tissue and control C0 were obtained randomly, by a blinded examiner, with 30x, 100x, 200 × and 1000 × magnification. The energy of the electron beam was 20 kV.

### PMMA wear assessment

Condylar and temporal acrylic components were digitized using a laser scanner (Tecnodrill, Digimill 3D, Brazil) with a conoscopic sensor (Optimet, ConoProbe 1000, Israel) and a 75mm lens and 0.05 mm resolution, creating a point cloud that was later transformed into a 3D mesh. Employing Geomagic Qualify software (Geomagic, USA), acrylic pieces removed from the animals were overlapped on control pieces (C0) – which represent the initial volume of the acrylic reconstruction – using manual and software alignment tools such as Best Fit Alignment. Image subtraction allowed observation of the linear wear (loss of height), in mm, by calculating the mean linear loss at three random points with the highest wear, as well as the loss of volumetric content of the material, in mm^3^, by calculating the volume difference from the wear plane in test samples in comparison to the control sample, during the postoperative experimental period of 45 days in both condylar and temporal samples. A blinded examiner performed the analyses.

### PMMA microhardness assessment

The condylar and temporal acrylic components removed from the animals, as well as a control sample (C0), were positioned and fixed with the use of brown Godiva on resin blocks. The Knoop hardness of the acrylic resin was evaluated using an HMV-2 automatic micro durometer (Shimadzu, Japan) with a load of 25 g for ten seconds. Three random indentations were performed on the surfaces (with and without wear) of each specimen, 100 μm apart. The final microhardness value assigned to each surface (with or without wear and control) on the condylar and temporal samples was the arithmetic mean of the three measurements. A blinded examiner conducted the analyses.

### Prosthetic stability

For analysis of macroscopic stability, the reconstructed TMJ was surgically exposed post-mortem for immediate evaluation of the retention of the acrylic structures. The presence of macroscopically observable movements of the prosthetic component in relation to the corresponding bone segment when the prosthesis was handled was taken to indicate a lack of prosthetic stability. The test was carried out with surgical instruments and manual pressure in lateral-medial directions, perpendicular to the direction of the force vector exerted by the reconstructed mandibular condyle on the reconstructed temporal surface. The examiner was blind to the time of death of the animals.

For radiographic evaluation, the left and right temporomandibular joints were removed post-mortem with a 6.0 cm (diameter) × 4.5 cm (depth) trephine drill and stored in enclosed containers with buffered formalin (10%). Within seven days following euthanasia, lateral X-ray examinations were used to examine the left TMJs.

The characterization of reconstructed mandibular and temporal structures as stable or unstable based on imaging examinations corroborated the results of the macroscopic analyses (T2). The experimenters were blind to the time of animal death.

### Functional evaluation

Maximum amplitude of mouth-opening and bilateral mandibular lateral movements were measured in millimetres using an optical pachymeter at three different times (T0, T1 and T2). The edges of lower central incisors and the upper edentulous alveolar ridges were used as reference for the measurement of the maximum mouth-opening amplitude. Since sheep do not have upper anterior teeth, the upper and lower labial frenums were used to measure the maximum amplitude of lateral movements. Mouth-opening and lateral movements amplitude were assessed through the application of a standard force of 2.5 kg, which was measured using a portable electronic scale. The same blinded examiner assessed the animals following the induction of muscle relaxation by carrying out the same measurements after obtaining a 1.5 MAC (minimum alveolar concentration) under isoflurane sedation.

### Body weight evaluation

Animals were weighed at the experimental unit, using a digital scale. The weight was recorded at two different times (T0 and T2). The examiner was blind to the animal group.

### Statistical analysis

Data were analysed using SigmaPlot 12.0 (Systat Software Inc., EUA), except for prosthetic stability, and functional and body weight evaluation, which were assessed using SPSS. The normality of data distribution was determined using the Shapiro–Wilk test, and variables were expressed as mean, standard deviation and median. A T-test for independent samples was applied to compare experimental groups for the number of inflammatory cells at different experimental times, and to compare volumetric and height wear between the experimental and control groups. Changes in the mean value of variables over time were compared within groups through a repeated-measures analysis of variance (functional measurements) and through paired-sample Student’s t-tests (weight). Variables were compared between the test and control groups by calculating deltas (differences between mean values at different points in time) and comparing these values between the groups using a one-way analysis of variance (ANOVA) followed by a Tukey post hoc test. A significance level of 5% was considered.

## Results

One sheep from the 90-day postoperative experimental time group presented with a series of systemic post-traumatic complications related to the attack of other animals in the field, during the housing period. However, as it survived until euthanasia, it was not excluded from the histological analysis. One of the samples of the temporal acrylic components presented dimensional deformation, probably due to mechanic compression in the trans-surgical acrylic preparation phase, preventing the analysis of linear and volumetric wear due to the impossibility of overlapping the scanned image with the one from the control sample. This feature might represent a limitation of the method used, but not of the technique itself.

### Histological assessment: pseudo-disc

**Fig. 2:**
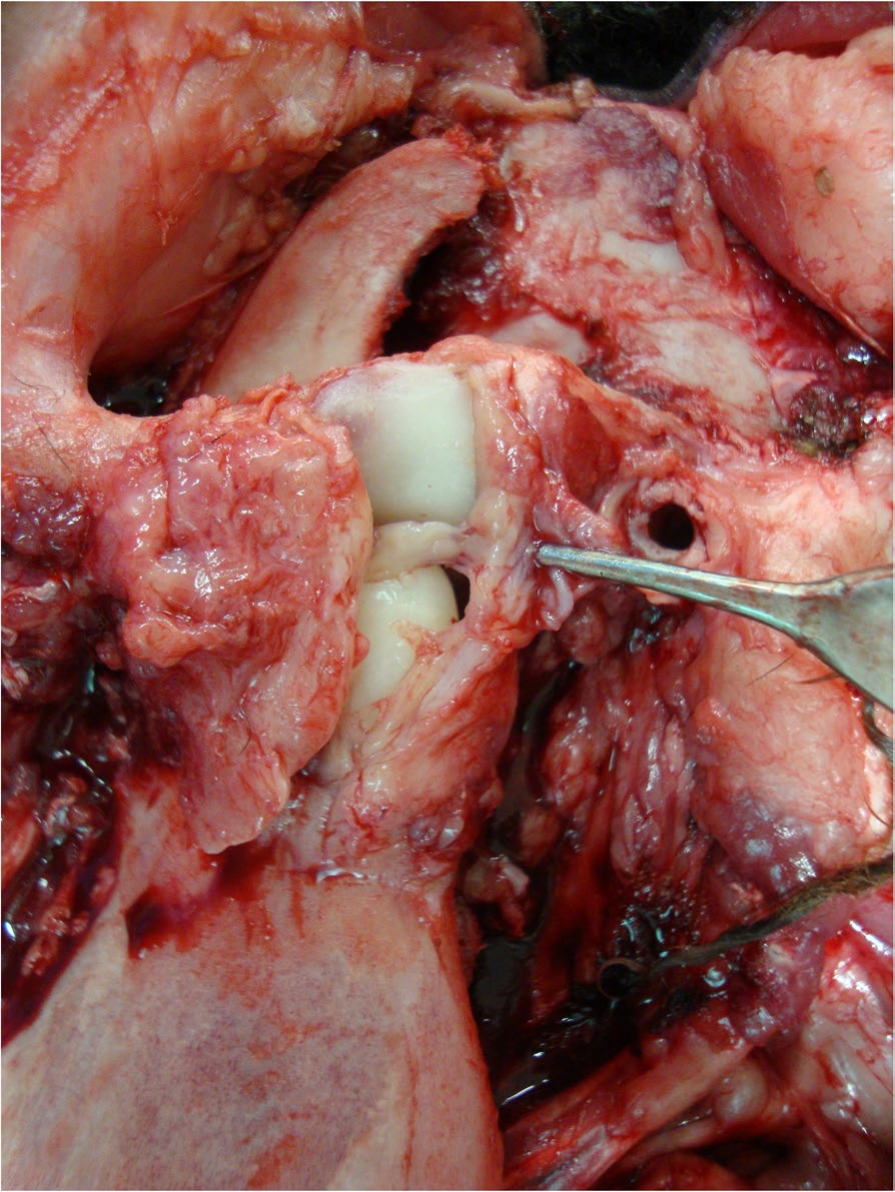
Post-mortem TMJ exposure and collection of peri-articular soft tissue samples. The pseudo-disc is macroscopically observed between the temporal and mandibular surfaces of the prosthetic reconstruction

A pseudo-disc was found in 90% of the joints, interposed between the temporal and condylar surfaces of the reconstruction (Fig. 2). Only one sheep, from the E90 group, did not present this tissue between the temporal and condylar PMMA reconstructed surfaces.

**Fig. 3:**
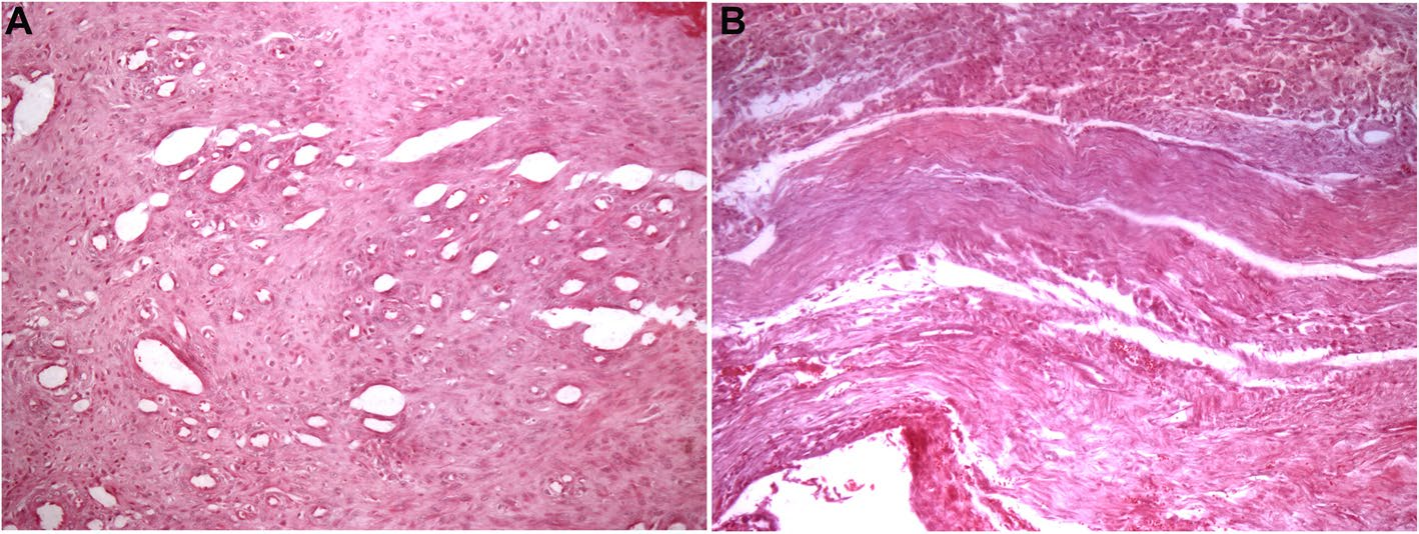
Pseudo-disc histological images. **A** Pseudo-disc at 45 days showing a highly cellularized and vascularized connective tissue (HE, 200x); **B** Pseudo-disc at 90 days showing a highly fibrous and oriented connective tissue (HE, 200x)

At 45 days (Fig. 3A), the samples of pseudo-disc presented a fibrous tissue with the presence of fusiform and bulky cells consistent with young fibroblasts, in addition to intense angiogenesis and vascular neoformation. A discrete inflammatory infiltrate composed mainly of lymphocytes and neutrophils was also observed. The presence of a fibrocartilaginous tissue was observed in the periphery.

At 90 days (Fig. 3B), this pseudo-disc became more fibrous, increasing the density of collagen fibres compared to 45 postoperative days. Fibroblasts became more mature, and many fibrocytes were observed. The tissue remained highly vascularized but was composed of more mature vessels. Inflammatory infiltrate decreased, and macrophages were observed in a few cases. The presence of a fibrocartilaginous tissue in the periphery was still observed.

As presented in Table 1, a numerical comparison of inflammatory cells between the 45th and 90th day postoperative experimental groups showed an 84% reduction in the number of lymphocytes over time (*p* < 0.05).

**Table 1:** Inflammatory cells quantification along the postoperative follow-up

**Disc**	**Lymphocytes**	**Neutrophils**	**Macrophages**
45 days	5.2 (± 5.187)	2.9 (± 4.32)	2.2 (± 3.41)
90 days	0.76 (± 1.20)	0 (± 0)	2.48 (± 6.09)
*p*	0.0002	NA	0.8876
**Capsule**	**Lymphocytes**	**Neutrophils**	**Macrophages**
45 days	2.76 (± 3.47)	0.4 (± 0.70)	1.44 (± 3.53)
90 days	1.3 (± 1.75)	0 (± 0)	1.52 (± 3.19)
*p*	0.037	NA	0.465

Data is presented as mean ± standard deviation and compared using test for independent samples

### Histological assessment: capsule

**Fig. 4:**
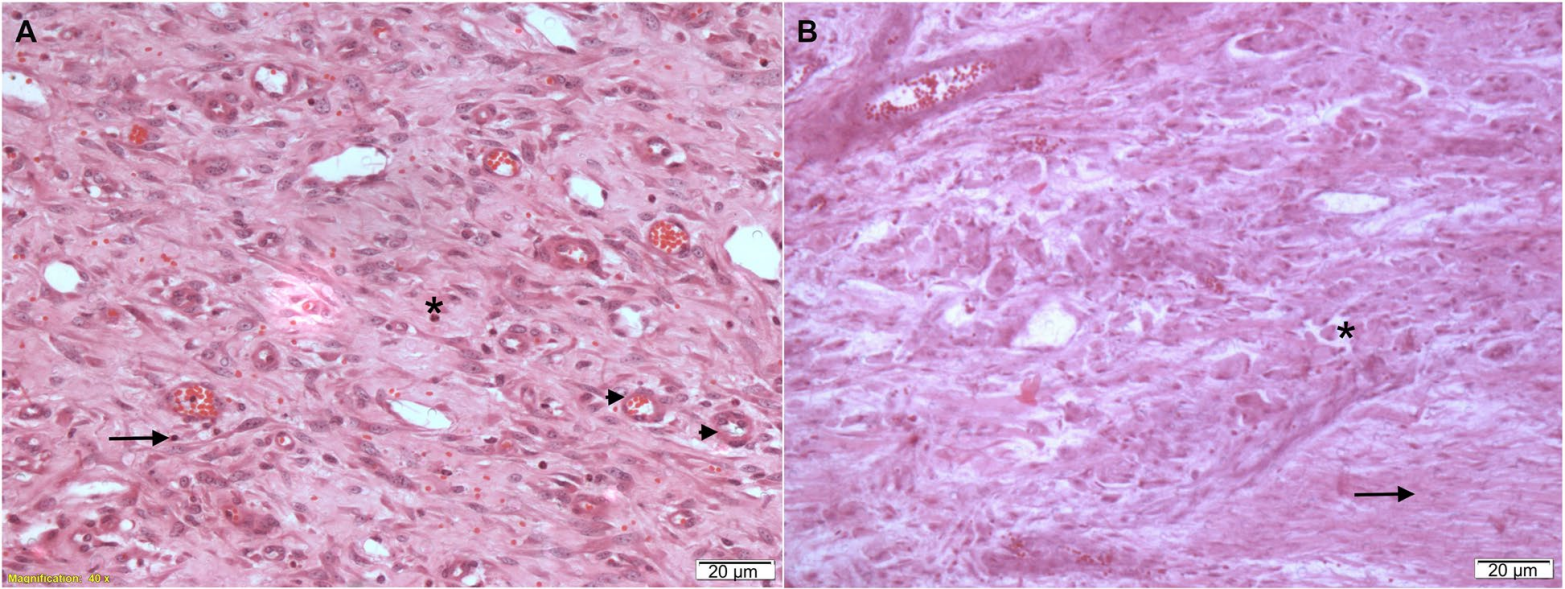
Histological capture from the reconstruction capsule showing inflammatory cells at different postoperative times. **A** 45-day postoperative experimental day. Asterisks, neutrophils; arrows, lymphocytes; arrowheads, angiogenesis; **B** 90-day postoperative day: asterisks, macrophages; arrows, fibroblasts and collagen fibres. Bar, 20 μm. HE staining

At 45 days, the reconstruction capsule showed fibrous connective tissue composed of young fibroblasts with foci of inflammatory infiltrate containing lymphocytes, macrophages and neutrophils (Fig. 4A). Samples were richly vascularized and contained areas of cartilaginous tissue. Discrete foci of ossification were observed.

At 90 days, fibrous connective tissue with mature fibroblasts and an absence of inflammatory infiltrate could be seen (Fig. 4B). Cartilaginous tissue with central ossification was also detected.

A numerical comparison of inflammatory cells between the 45th and 90th day postoperative experimental groups showed a 100% reduction in the number of neutrophils over time (*p* < 0.05) (Table 1).

### PMMA degree of conversion

**Fig. 5:**
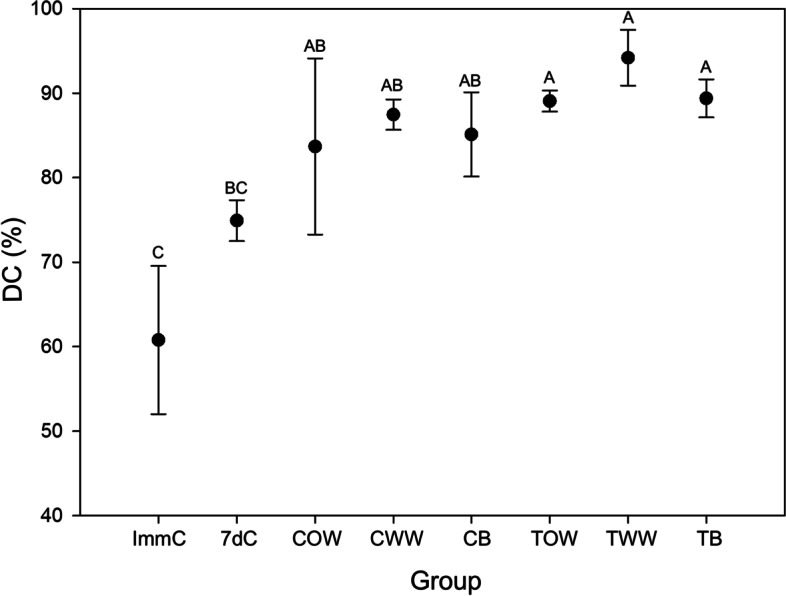
Comparison of the degree of conversion (DC) among test and control groups. ImmC (immediate control), 7dC (7-day control), COW (condylar without wear), CWW (condylar with wear), CB (condylar in contact with bone), TOW (temporal without wear), TWW (temporal with wear), TB (temporal in contact with bone). Data are presented as mean and standard deviation, and were compared using an analysis of variance (ANOVA) followed by Tukey’s post hoc tests. Different capital letters represent the presence of statistical differences (*p* < 0.05)

Condylar and temporal samples showed a similar degree of conversion of the alloplastic material when surfaces with and without wear were compared, or when considering the areas of contact with bone tissue (Fig. 5). Condylar surfaces with wear, without wear and area of contact with the bone presented the same degree of conversion as the seven-day control group. On the other hand, temporal surfaces showed a higher degree of conversion (*p* < 0.05) than immediate and seven-day control groups. The immediate control group presented the same degree of conversion as the seven-day control group, but this value was lower than in all test groups (*p* < 0.05).

### Surface roughness

Non-wear surfaces of condylar components showed higher roughness values than both control surfaces and those in other test groups (*p* < 0.05) (Table 2). Temporal non-wear surfaces showed no difference compared with the control group, but presented a greater roughness (*p* < 0.05) than temporal and condylar surfaces with wear. These same surfaces with wear presented lower surface roughness than both the control group and non-wear temporal and condylar surfaces (*p* < 0.05).

**Table 2:** Comparison of the surface roughness (µm) among test and control groups

Group	Surface roughness (µm)
Control	1.56 (± 0.61) B
Condylar without wear	2.67 (± 0.57) C
Condylar with wear	0.17 (± 0.09) A
Temporal without wear	1.55 (± 0.08) B
Temporal with wear	0.11 (± 0.04) A

Data is presented as mean ± standard deviation and compared using an analysis of variance (ANOVA) followed by Tukey post-hoc tests. Different capital letters represent the presence of statistical differences (*p* < 0.05)

### Scanning electron microscopy

**Fig. 6:**
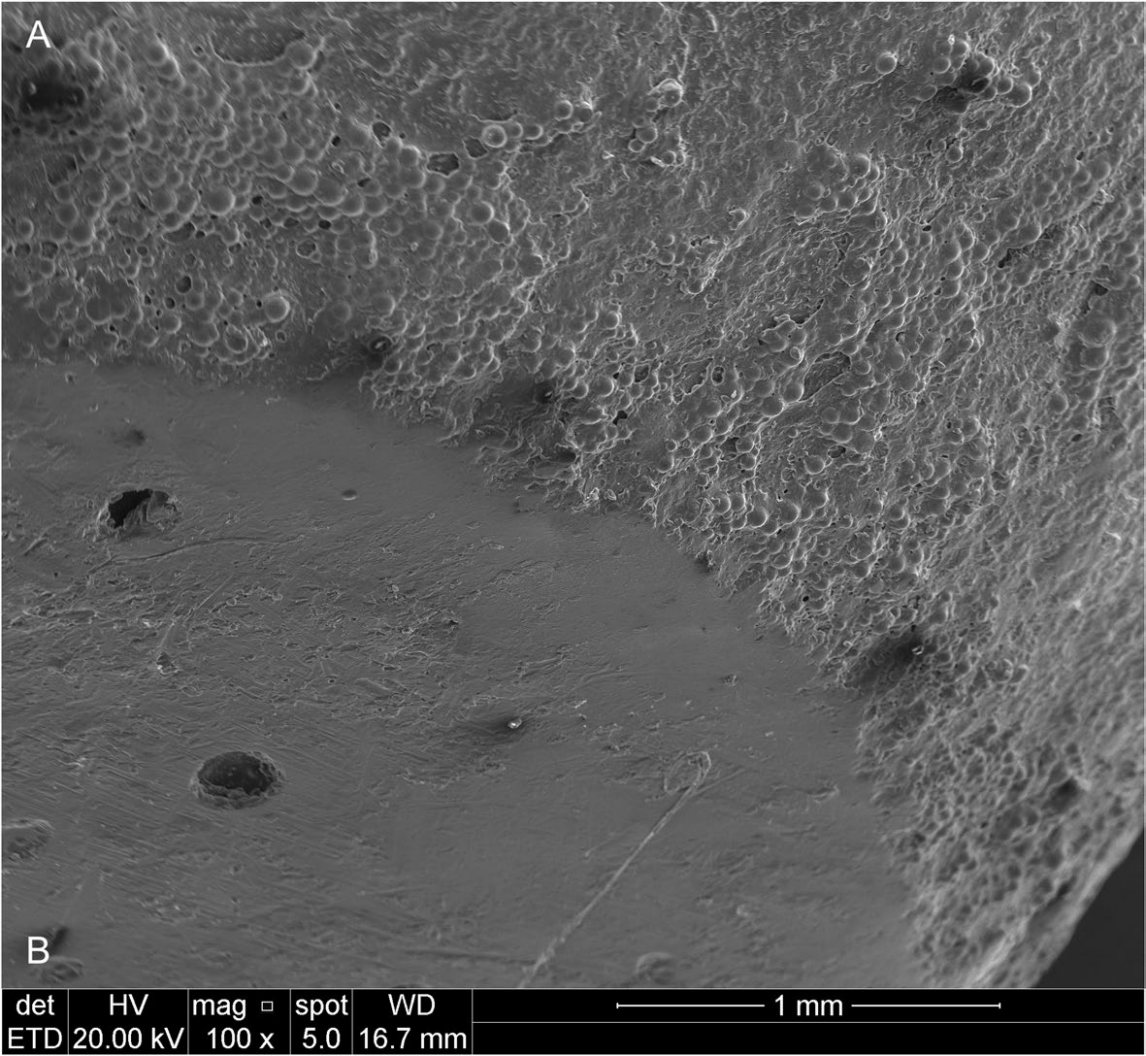
Scanning electron microscopy (100x) – image from PMMA temporal component showing the different pattern of porosity in surface of articular contact. **a** Surface without wear; **b** Surface with wear, compatible with the articular contact area

SEM images enabled surface characterization of the acrylic components, in which a difference in the porosity pattern of the samples was observed (Fig. 6). A specific and well-demarcated surface of articular contact was identified in all samples from the test group. These articulating surfaces, already observed macroscopically, had indeed a lower surface porosity than the other random surfaces when observed microscopically.

### PMMA wear

The linear wear (height loss) of mandibular components (0.55 mm ± 0.17 mm) of the reconstruction region showed no statistical difference in comparison with the control group. Temporal components presented no difference in linear wear compared with mandibular components; however, they presented higher wear (0.76 mm ± 0.19 mm) than the control group (*p* < 0.05).

**Fig. 7:**
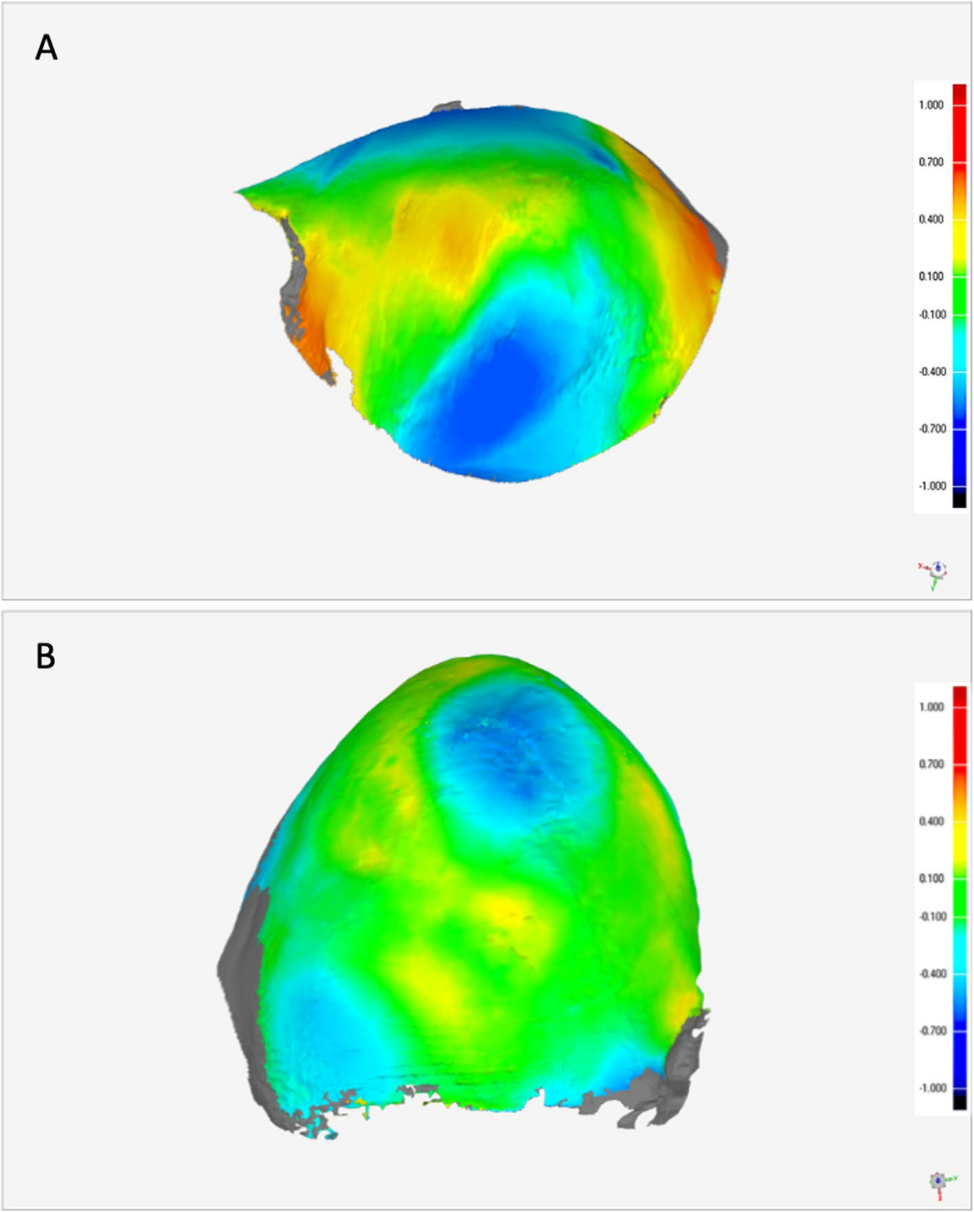
Overlapping of the volumetric reconstructions of the temporal (**A**) and mandibular (**B**) acrylic components to the corresponding control samples. Red represents a larger volume (mm^3^) while blue represents a smaller volume (mm^3^) of the test component in comparison to the control sample

Mandibular components (6.54 mm^3^ ± 2.25 mm^3^) of the reconstruction did not present statistical difference in volumetric wear in comparison with the control group (*p* > 0.05). Temporal components showed higher volumetric wear (18.56 mm^3^ ± 6.89 mm^3^) than mandibular components and also compared to the control group (*p* < 0.05) (Fig. 7).

### PMMA microhardness

No statistically significant difference was observed in the comparison of the Knoop hardness (KHN) among control and test groups in temporal and condylar components, respectively, on surfaces with (17.71 ± 2.78; 18.56 ± 2.19) and without (21.11 ± 8.34; 18.56 ± 2.19) wear.

### Prosthetic stability

**Fig. 8:**
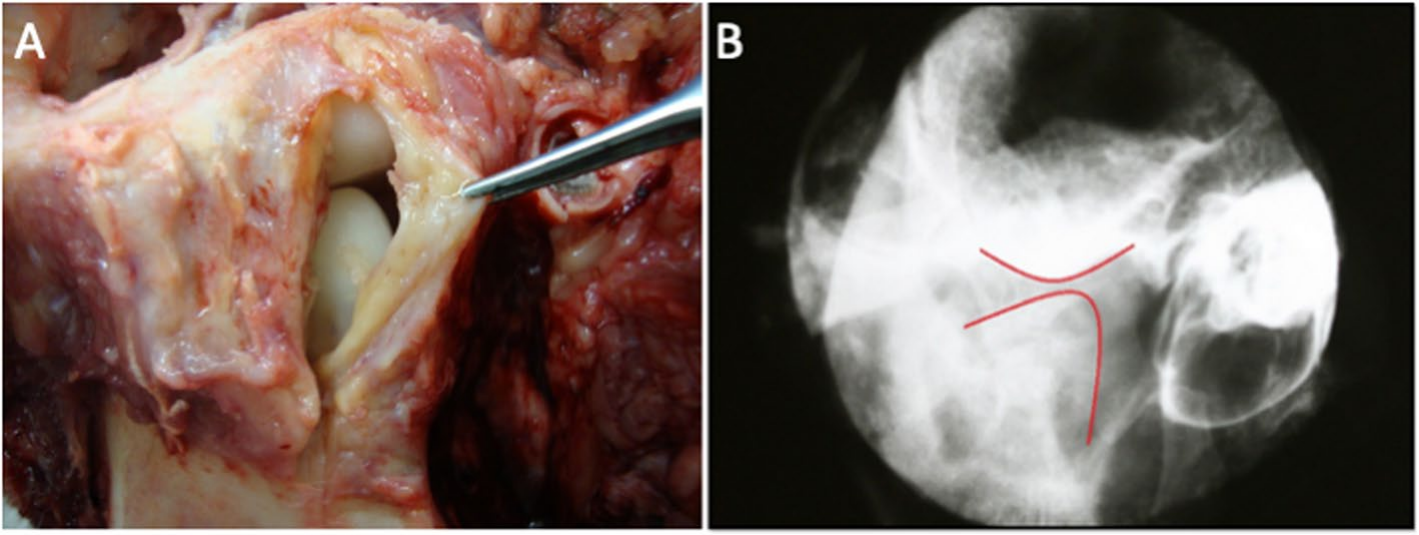
Macroscopic and imaging examinations in the postoperative period. **A** Post-mortem TMJ exposure and collection of peri-articular soft tissue samples. The image shows the clamping of the capsule, which covers the joint reconstruction for the evaluation of reconstruction stability; **B** Indication of the articular reconstruction site in the X-ray image

After necropsy, both segments of the articular reconstructions were found to be macroscopically stable in experimental group animals (E45 and E90) (Fig. 8A).

The radiographic images confirmed the accurate placement of the prosthetic structures and the absence of displacement, which had already been clinically observed post-mortem (T2) in all animals. In Fig. 8B, the red lines represent the articular surfaces of the new joint.

### Functional evaluation

**Fig. 9:**
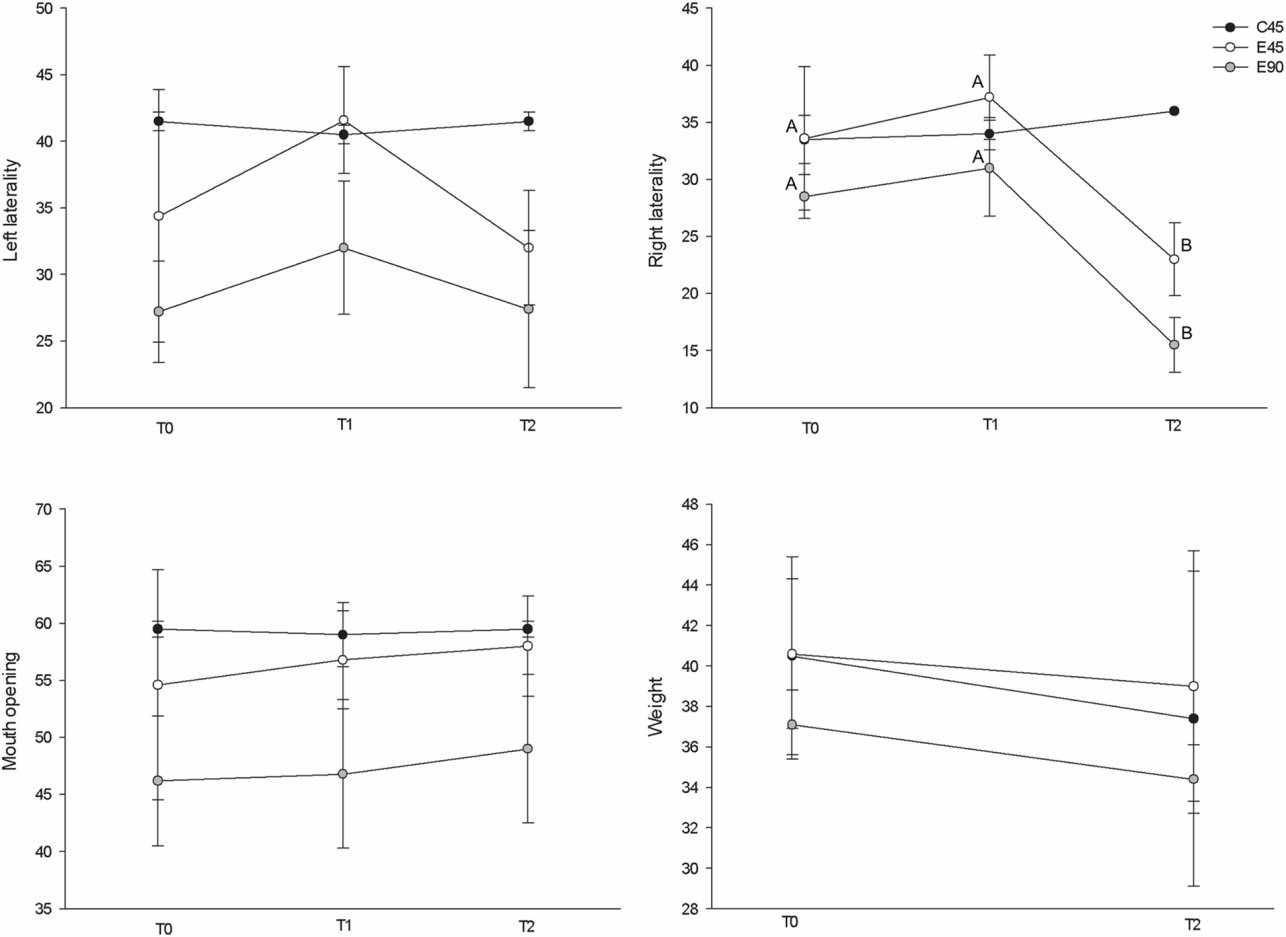
Comparison between mean maximum mouth-opening amplitudes, left and right lateral movements and animal body weight (in millimetres and kilograms, respectively). For the variable weight the sample size of group E90 was *n* = 4. Variables are expressed as mean ± standard deviation. Within-group comparisons were performed through repeated-measures analysis of variance (at 3 different moments) or paired-sample T-tests (weight). Letters (A, B) represent significant statistical differences

As can be seen in Fig. 9, in the C45 group no statistical differences were observed over time in any of the three functional variables evaluated. In the E45 and E90 groups, there were no statistical differences in the maximum amplitude of mouth-opening and left lateral movements between the three times of assessment (T0, T1 and T2). However, the range of right lateral movements differed (*p* < 0.001) between T0 and T2, as well as between T1 and T2.

There were no significant differences among any of the groups (E45, C45 and E90) in terms of mouth-opening measurement deltas (T1–T0 and T2–T0). Similar results were obtained for the amplitude of left lateral movements. The difference between the amplitude of right lateral movements at T2 and T0 differed between the E45 and C45 groups, as well as between the E90 and C45 groups (p < 0.005). No significant between-group differences were observed in the T1–T0 delta (Table 3).

**Table 3:** Comparison of the differences between mean maximum mouth opening amplitude, left laterality, right laterality, and weight (in millimeters and kilograms) between experimental and control groups over the course of the study

Variable	Delta (post—pre)	C45 *n* = 2	E45 *n* = 5	E90 *n* = 5*	P
Mouth opening	T1—T0	-0.50 ± 2.12 (-0.50)	2.20 ± 6.72 (-2.00)	0.60 ± 5.59 (-1.00)	0.891
	T2—T0	0.00 ± 0.00 (0.00)	3.40 ± 6.58 (2.00)	2.80 ± 5.81 (5.00)	0.838
Left laterality	T1—T0	-1.00 ± 0.00 (-1.00)	7.20 ± 7.19 (5.00)	4.80 ± 2.39 (5.00)	0.133
	T2—T0	0.00 ± 1.41(0.00)	-2.40 ± 8.73(-2.00)	0.20 ± 6.46 (1.00)	0.942
Right laterality	T1—T0	0.50 ± 0.71 (0.50)	3.60 ± 4.45 (5.00)	2.50 ± 4.65 (3.50)	0.791
	T2—T0	2.50 ± 2.12^b^ (2.50)	-10.60 ± 4.67^a^ (-12.00)	-13.00 ± 3.56^a^ (-14.00)	0.004
Weight	T2—T0	-3.10 ± 3.39 (-3.10)	-1.58 ± 2.72 (-1.10)	-2.65 ± 2.46 (-2.35)	0.872

Data is presented as mean ± standard deviation (median) and compared using an analysis of variance (ANOVA) followed by Tukey post-hoc tests. Different letters represent the presence of statistical differences (*p* < 0.05)

^*^For the variable weight, the sample size of group E90 was *n* = 4

^**^A post-hoc test was not possible due to a lack of variability (*n* = 1)

### Body weight evaluation

There were no statistically significant differences in average weight in the pre-operative period (T0) or in the postoperative period (T2) in the C45, E45 and E90 groups (Fig. 9). When the T2–T0 delta was calculated, between-group comparisons showed no statistical differences, suggesting an absence of differences between experimental and control groups in the variation between pre- and postoperative weight (Table 3).

## Discussion

End-stage temporomandibular joint pathology requires TMJ reconstruction, which can be accomplished with autogenous tissue or alloplastic materials [23]. Puricelli biconvex arthroplasty technique enables the reconstruction of this joint using two PMMA convex surfaces. The technique has been successfully used for over 20 years, with long-term positive results [16–18, 24]. In the present study, we investigated the functional outcome of the procedure as well as maintenance of physical properties of the material in a sheep model of the ABiP method.

The use of sheep models in research involving disease induction [25], diagnosis and treatment [26–28] or TMJ reconstruction techniques [21, 29] is justified by the dimensional, structural and morphological similarities between sheep and human joints. The constant and intense chewing activity of ruminants has also been suggested in the literature as a benefit of this animal model [30]. According to Matsuura et al. [26], evaluations conducted within a short postoperative period (90 days) should be sufficient for determining the effects of TMJ reconstruction on sheep jaw function, and are equivalent to long-term evaluations in humans. In our study, the 45-day period was adopted based on the data from an experimental study in rabbits [31], which involved the reorientation of the force vector of the jaw to the base of the skull, a principle similar to that on which the biconvex arthroplasty technique is based [24, 32]. The histomorphometric analysis of the temporal bone of the animals suggested increased bone remodelling rates during the evaluated period, which is consistent with our clinical observations in humans [16–18, 24].

In 45 and 90 postoperative days, respectively, the results showed macroscopic and radiographic stability of the acrylic components in all the animals. For Kummoona [33], the use of auto-polymerized poly(methyl methacrylate) leads to improved adaptation of the implant to the bone and reduces the stress on the joint, transferring the masticatory load to the marrow bone. These features may have contributed to the lack of displacement of the prosthetic devices.

Having confirmed the stability of the reconstruction using biconvex arthroplasty, we sought to understand the ability of the PMMA in this technique to support TMJ functional loads. In our study, an area of greater polishing was observed on the surface of each acrylic component after removal from the mandibular and temporal bone tissue, compatible with the point of contact between them, as proposed in the original technique. The surface roughness analysis showed that these areas of contact between the articular components did indeed have a lower roughness than control samples and other areas evaluated in the same components, suggesting the loss of superficial content (wear) through friction of the acrylic surfaces due to joint movements.

Scanning electron microscopy images showed a difference in the pattern of surface porosity when comparing articular contact surfaces with other random surfaces of the acrylic components. SEM images also made it possible to identify a specific and well-defined surface of articular contact in each sample, which corresponds to the original descriptions by Puricelli [24, 32] of a single and minimum point of contact between the reconstructed surfaces, favouring mandibular movements. This contact surface showed lower porosity than wear-free surfaces and control samples.

The higher degree of conversion of acrylic resin observed in our seven-day control samples and in test samples than in immediate control suggests the maintenance of the polymerization process, albeit at a lower intensity, throughout this period. Numerically, the areas with wear presented higher values of the degree of polymerization, although without statistical difference, suggesting a possible influence of the heat generated by the friction between the surfaces during the chewing function, while the material still presented active polymerization. The high degree of conversion obtained in the seven-day and control samples from our study is similar to values described in the literature for PMMA [34]. These results favour a greater biocompatibility of the material, since the higher the degree of polymerization of the resinous material, the smaller the amount of free monomers that can be leached to the adjacent tissues. The presence of residual monomers in the surrounding tissues can cause not only a local inflammatory response, but also systemic allergic reactions [35].

Despite the differences in polymerization degree values, the samples did not present statistically significant variations in microhardness of the material in this study. Hardness and excessive abrasion resistance can be a disadvantage in TMJ implants. Due to the high modulus of elasticity, chromium-cobalt alloys used in TMJ metal prostheses cause significant stress shielding, which can lead to implant migration and bone fracture secondary to deep bone resorption [36]. Titanium alloys have half the modulus of elasticity of chromium-cobalt. This greater elasticity is able to transfer the functional loads more evenly, causing less stress shielding to adjacent bone tissue [36]. However, titanium does not have good properties when it comes to wear. When submitted to repetitive forces, its fatigue can lead to fracture [36]. These properties explain the use of titanium as base, while chromium-cobalt is used as the joint surface of the mandibular components in the current metallic prostheses.

In orthopaedics, hip prostheses using chromium-cobalt and ultra-high-molecular-weight polyethylene (UHMWPE) have shown good resistance to wear; however, in TMJ, the functional loads are relatively high and the area of articular contact is smaller, which is a dynamic that favours a more aggressive wear due to increased contact stress [36]. UHMWPE has been used as a joint surface in the temporal prosthetic component, but has shown wear, with particularization, osteolysis and loss of stability [36]. Metal-on-metal chromium-cobalt prostheses have been shown to have low wear rates compared to polyethylene. However, they release particles and ions into the adjacent tissues and systemically, especially shortly after implantation. These metallic ions, especially chromium, are possible cytotoxic and carcinogenic agents, also related to allergic reactions [36].

In this study, mandibular acrylic components did not present a loss of height or volume in comparison with control samples. Temporal and mandibular components showed the same height loss, but temporal components presented a statistically significant loss of height (0.76 mm ± 0.19 mm) compared with the control sample. Temporal components also presented greater volumetric loss (18.56 mm^3^ ± 6.89 mm^3^) than mandibular and control samples. The loss of height and volume could lead to impairment in mandibular movements, but no changes in the range of the mandibular movements or interference in feeding (by evaluating body weight) were observed in the experimental periods of 45 and 90 days following biconvex arthroplasty, suggesting that the magnitude of wear observed in our samples does not have relevant clinical repercussions.

Another concern regarding wear refers to fragmentation and particulation of the material with deposition in adjacent tissues [37], which may lead to resorption of supporting bone tissue and inflammatory foreign body reaction [37–39]. Poly(methyl methacrylate) has been reported to have biocompatibility [24, 32, 40–42], and these results were corroborated by the present findings. The number of inflammatory cells showed a reduction from the 45-day to the 90-day experimental period. Histological analysis also confirmed the absence of foreign body reaction in the tissues adjacent to the alloplastic reconstruction, despite the observed wear. These findings, associated with the absence of signs of infection or of the rejection/expulsion of the material in the animals involved in the present study, are suggestive of biological compatibility between the PMMA and organic tissues in the follow-up period. Even the sheep that presented a hind limb local infection displayed no signs of infection in the TMJ area.

Both subjective and objective criteria for the assessment of the success of TMJ reconstruction procedures in humans have been suggested in the literature. Some of the subjective criteria proposed can be assessed by means of a visual analogue scale, and include pain, jaw function and diet [43]. The objective criteria, which are evaluated by physical measurement, include the maximum interincisal [21, 44, 45] and lateral mandibular movements [21, 29]. In the present study, no statistical differences were observed in the measurements of mean amplitude of mouth-opening and left lateral movements taken in the pre-operative, immediate postoperative and postoperative periods in animals subjected to TMJ reconstruction. The amplitude of right lateral movements in the experimental group was significantly lower in the late postoperative period than in the other two assessment periods (preoperative, postoperative). No statistical differences were observed between any of the measurements of the amplitude of mouth-opening taken in experimental groups and control animals. The same pattern of results was found for the amplitude of left lateral movement. The amplitude of the change in right lateral movements between the late postoperative period and the pre-operative period, however, differed significantly between animals submitted to TMJ reconstruction and the animals in the control group. Matsuura et al. [26] induced ankylosis in the right TMJ of sheep and evaluated the effect of gap arthroplasty in these animals. The authors found an increase in movement amplitude in the immediate postoperative period, which was not maintained after 90 postoperative days. However, there was still a statistical difference between the amplitude of the three movements evaluated when measurements taken in the early (physiological articulation) and late (90 days post-arthroplasty) experimental period were compared.

The reduced amplitude of right lateral movements found in the present study may have been caused by the formation of fibrous tissue around the reconstruction site, which may have limited the movement of the new joint. However, contralateral movements showed no such limitations. Additionally, the detachment and subsequent atrophy of the left lateral pterygoid muscle may have promoted a decrease in contralateral jaw movements, since it plays an important role in lateral movements as a whole.

The literature has also discussed the effects of TMJ mobilization following reconstructive surgery [40, 46]. According to Puricelli [40], biconvex arthroplasty enables the early mobilization of the TMJ in the postoperative period. The consequences of early mobilization of the TMJ in the postoperative period following the removal of maxillomandibular immobilization, and the possibility of bilateral indication, must also be seen as important advantages of this technique [47]. In the present study, the animals were returned to their normal diets in the immediate postoperative period, without the need for physical therapy or dietary restrictions. It is important to note that the animals showed no pathological changes such as TMJ ankylosis prior to reconstruction. The absence of muscle atrophy may have favoured the use of early mobilization in the animals in the present study.

TMJ ankylosis is described in the literature as one of the most common complications of surgical joint interventions [48]. In the present study, none of the animals submitted to biconvex arthroplasty presented with this condition. Since our purpose was to evaluate the reproducibility of the technique and its functional outcome, no such pathologies were purposely induced. Fibrocartilage observed in the periphery of the pseudo-disc samples could be related to remaining tissue from the original joint disc.

Weight alterations reflect changes in the balance between nutrient intake and consumption [49]. Comparisons between the pre- and postoperative weight of animals undergoing left TMJ reconstruction revealed no statistical differences in these values, showing that the animals had no significant weight loss. The same finding was observed in the 45-day control group. The variations in the weight of animals submitted to joint reconstruction were also statistically similar to those observed in control group animals. Matsuura et al. [26] observed no statistical difference in animal weight between the period prior to the induction of ankylosis and measurements taken 90 days after this point and 90 days after an arthroplasty. Cheung et al. [21] also observed no significant reductions in animal weight over the course of the experimental period.

PMMA cement has been widely used for bone reconstruction purposes [50]. Although, as presented above, concerns have arisen about possible risks in using this polymer, current alternatives allow its safe use in different medical specialties (reviewed by Gergely et al. [12]). Our experience with the ABiP technique has shown clinical success including in long-term follow-ups, stimulating the pursuit, understanding and production of evidence regarding the efficacy of this reconstruction technique [16–18, 24]. It is important to highlight that the use of plastic PMMA for TMJ reconstruction in Puricelli biconvex arthroplasty differs from that described in previous literature, in which the acrylic parts are assembled prior to insertion at the surgical site [23, 51].

There are some limitations to be addressed in this study, mainly related to the use of a large animal model. A larger number of animals, such as seen in rodent models, is more difficult due to costs and maintenance constraints. Image exams were done in TMJ samples removed post-mortem, and we acknowledge that imaging of live animals could be more informative.

The criteria used to assess the success of TMJ reconstruction procedures are the stability of the reconstructed structure, its capacity to bear functional load and the biocompatibility of the alloplastic material [36, 46, 52]. According to these criteria suggested by Mercuri [46, 52], the present study has produced clinical, histological, material-related and radiographic evidence of the successful applicability of biconvex arthroplasty for TMJ reconstruction.

## Conclusions

The maintenance (or recovery, when a pathology is present) of joint mobility and body weight is considered to be indicator of TMJ function joint. The application of TMJ reconstruction using the Puricelli biconvex arthroplasty technique was found to produce stable results. Body weight and all mandibular movements, save for contralateral mandibular ones, were maintained. Minor superficial wear on the articular surface of PMMA components was observed, with regression of local inflammatory response over time and no foreign body in the studied sample. These data suggest that joint function was completely maintained following the procedure and emphasize the relevance of biconvex arthroplasty in TMJ reconstruction procedures.

## Data Availability

Datasets and further information are available by contacting the corresponding author.
